# Zahnmedizinische Beschwerdebilder mit psychosozialem Hintergrund

**DOI:** 10.1007/s00103-021-03369-y

**Published:** 2021-07-01

**Authors:** Anne Wolowski, Hans-Joachim Schneider, Thomas Eger

**Affiliations:** 1 Poliklinik für Prothetische Zahnmedizin & Biomaterialien, Zentrum für Zahn‑, Mund- und Kieferheilkunde, Universitätsklinikum Münster (UKM), Albert-Schweitzer-Campus 1, 48149 Münster, Deutschland; 2 Abt. XXIII Zahnmedizin, Parodontologie Bundeswehrzentralkrankenhaus Koblenz, Koblenz, Deutschland

**Keywords:** Zahnbehandlungsangst, Parodontitis, Funktionsstörungen, Okklusale Dysästhesie, Somatoforme Prothesenunverträglichkeit, Dental anxiety, Periodontitis, Functional disorders, Occlusal dysaesthesia, Somatoform denture intolerance

## Abstract

Obwohl Mund und Zähne lebenslang eine zentrale Bedeutung für die Entwicklung und das Wohlbefinden eines Menschen haben, finden psychosoziale Aspekte von Krankheit und Gesundheit nur zögerlich Eingang in zahnmedizinische Erklärungsmodelle. Als interventionsbedürftige Störung wird einzig die Zahnbehandlungsangst mit Krankheitswert im Sinne einer spezifischen Phobie allgemein anerkannt. Diese beschreibt die intensive Gefühlsreaktion auf Elemente der zahnärztlichen Behandlungssituation, die für den Betroffenen Leiden verursacht und die angesichts der tatsächlichen Gefahren in der Situation übertrieben erscheint. Ansonsten besteht jedoch nach wie vor die Tendenz, Beschwerden im zahnmedizinischen Kontext eher somatisch zu erklären. Das wirkt sich auf die Erwartungshaltung Betroffener wie auch auf die interdisziplinäre Zusammenarbeit aus. Zur Verbesserung der interdisziplinären Unterstützung und des gegenseitigen Verstehens stellt der folgende Beitrag neben der Zahnbehandlungsangst und deren Folgen für die Mundgesundheit weitere Beschwerde- und Krankheitsbilder vor. Bei kraniomandibulärer Dysfunktion, Bruxismus, okklusaler Dysästhesie sowie somatoformer Prothesenunverträglichkeit können psychosoziale Aspekte in Entstehung, Verlauf und Bewältigung ebenfalls eine bedeutende Rolle spielen.

## Abstract (en)

While the mouth and teeth play a lifelong central role in a person’s development and wellbeing, psychosocial aspects of disease and health are still only reluctantly included in dental explanatory models. Only dental anxiety with its disease quality of a specific phobia is generally recognized as a mental disorder requiring intervention. It is interpreted as an emotional reaction to aspects of dental treatment, which results in distress for the affected person and appears to be unreasonably intense given the actual dangers involved. Apart from that, the tendency to provide a somatic explanation for symptoms in the dental context persists. This bears implications for the expectations of those affected as well as for interdisciplinary cooperation. In order to improve interdisciplinary support and mutual understanding, the following article introduces the clinical pictures of craniomandibular dysfunction, bruxism, occlusal dysaesthesia, and somatoform prosthesis intolerance alongside dental anxiety. Psychosocial factors can profoundly influence the development, course, and management of these conditions.

## Einführung

Traditionell gilt die Zahnmedizin als primär somatisch orientiertes Fach. Somit ist es für PatientInnen schwer nachvollziehbar, wenn rein somatisch erlebte Symptome nicht bzw. nicht nur somatisch erklärt werden können und psychosoziale Einflussfaktoren eine Rolle spielen sollen. Diese können unabhängig von zahnmedizinischen Befunden wesentliche Co- oder auch Kausalfaktoren sein. Ein Nichterkennen solcher Zusammenhänge im Sinne des auch für die Zahnmedizin geltenden biopsychosozialen Krankheitsmodells hat fatale Folgen, die durch viele erfolglose, irreversibel schädigende und oft kostspielige Maßnahmen gekennzeichnet sind. Betroffene sind schließlich nicht mehr bereit, ihr eigenes rein somatisches Krankheitsmodell aufzugeben. Die Beschwerden chronifizieren, Wut, Aggression und gegenseitige Kränkungen enden vielfach in juristischen Auseinandersetzungen, weil Betroffene sich missverstanden und stigmatisiert fühlen. Sie wollen rehabilitiert werden und beweisen, dass ihr Leiden nicht psychosomatisch begründet ist, sondern sie „Opfer“ einer zahnärztlichen Fehlleistung sind. Diese Krankheitsverläufe lassen sich vor allem bei PatientInnen mit der Diagnose okklusale Dysästhesie (OD) und somatoforme Prothesenunverträglichkeit (SP) beobachten [1, 2]. Beide Krankheitsbilder müssen aufgrund einer vergleichbaren Symptomatik differenzialdiagnostisch abgegrenzt werden von funktionellen Störungen wie Bruxismus und der kraniomandibulären Dysfunktion (CMD), zumal auch bei diesen Erkrankungen psychosoziale Aspekte in Entstehung, Verlauf und Bewältigung eine bedeutende Rolle spielen [3, 4]. Während Betroffene bei steigendem Leidensdruck eher zu einem Verhalten im Sinne eines „Ärztehoppings“ [5] tendieren, vermeiden ca. 5–10 % der Bevölkerung aufgrund einer Zahnbehandlungsangst (ZBA) die zahnärztliche Untersuchung und Therapie. Ein damit oft einhergehend zunehmend desolater Zahn- und Parodontalbefund treibt Betroffene mehr und mehr in die soziale Isolation. Zusammenhänge werden oftmals nicht oder zu spät erkannt [6].

Grundsätzlich muss jedoch auch bedacht werden, dass das Nichterkennen von somatischen (möglicherweise wenig offensichtlichen) Befunden dazu führen kann, dass PatientInnen einen hohen Leidensdruck aufbauen und auf diesem Wege psychosomatisch auffällig werden. Grundlage für die frühzeitige Vermeidung solcher Krankheitsverläufe ist eine umfassende, nicht redundante somatische und ggf. gezielt interdisziplinäre Diagnostik. Diese erlaubt die Beurteilung, ob eine Diskrepanz zwischen objektivierbaren Befunden und der subjektiv erlebten Befindlichkeit besteht. Nur so lässt sich das Ausmaß der psychosomatischen Mitbeteiligung feststellen. Eine enge Kooperation von Zahnmedizin und der im Einzelfall relevanten Fachdisziplin(en) muss getragen sein von gegenseitigem Verständnis und insbesondere für die Betroffenen transparent und nachvollziehbar gestaltet sein.

Die hohe Relevanz des Wissens um psychosomatische Aspekte im Kontext zahnmedizinischer Maßnahmen ergibt sich zudem aus der Tatsache, dass mindestens 20 % aller PatientInnen über derartige Beschwerden klagen und man im Unterschied zu sonstigen FachärztInnen davon ausgehen kann, dass ZahnärztInnen ähnlich wie HausärztInnen PatientInnen entsprechend des Querschnitts der Bevölkerung sehen [7].

Im folgenden Beitrag werden die wesentlichen Merkmale, diagnostische und therapeutische Aspekte psychosozial beeinflusster Krankheitsbilder im Bereich der Zahnmedizin dargestellt.

## Zahnbehandlungsangst (ZBA)

Die Häufigkeit von ZBA mit Krankheitswert liegt bei 5–10 % der Bevölkerung [8]. Angst- und depressive Störungen sind häufiger bei Menschen mit ZBA mit Krankheitswert zu finden [9]. ZBA ist ein wenig beachtetes Symptom in Zusammenhang mit posttraumatischer Belastungsstörung [6], Parodontitis [10] und mit eingeschränkter mentaler Gesundheit [11]. Nach Lenk et al. [11] weist ein Drittel der PatientInnen mit einer bekannten psychosomatischen Krankheit eine stark erhöhte ZBA auf.

Die Entwicklung von ZBA mit Krankheitswert gilt als multifaktoriell und zählt zu den spezifischen Phobien, welche charakterisiert sind durch intensive Angst bei zahnärztlicher Behandlung oder Vermeidung derselben [8]. Die spezifische Phobie zeichnet sich durch eine unangemessene Angstreaktion bei Vorliegen von eindeutig definierten, eigentlich ungefährlichen Stimuli aus. Der Einfluss traumatischer Erfahrungen, familiärer Beeinflussung und individueller Charaktereigenschaften ist gut belegt [6, 8–10].

### Diagnostik der ZBA

ZBA behindert die erfolgreiche zahnärztliche Behandlung. Da eine gewisse Angst vor der Zahnbehandlung als normal gilt, unterbleibt vielfach im zahnärztlichen Routinebetrieb eine gezielte (Differenzial‑)Diagnostik der ZBA. In jedem Fall sollten vegetative und allgemeine Symptome sowie angsttypisches Verhalten (z. B. Meiden des Blickkontaktes, zögerliches Antworten, Schreckreaktionen) wahrgenommen und thematisiert werden. Visuelle Analogskalen zur Selbsteinschätzung einer ZBA sind sinnvolle Diagnosehilfen ebenso wie der hierarchische Angstfragebogen (HAF, bestehend aus 11 Fragen mit Antwortmöglichkeiten von jeweils „überhaupt nicht ängstlich“ bis „krank vor Angst“; [12]). Die Diagnose einer ZBA mit Krankheitswert ist hierbei erst gerechtfertigt bei einem Summenscore > 38 verbunden mit zahnmedizinischem Vermeidungsverhalten über mindestens 2 Jahre [13, 14]. Tab. 1 stellt die wichtigsten zahnärztlichen diagnostischen und behandlungsvorbereitenden Maßnahmen bei Betroffenen dar.

**Table 1:** 

Behandlungsvorbereitung bei Verdacht auf Zahnbehandlungsangst (ZBA)
*Anamnese, Fremdanamnese, Differenzialdiagnostik, Diagnosestellung und Schweregrad*
Selbsteinschätzung der Angst mittels visueller Analogskala (VAS) von 0–100 mm
Angst-VAS-Wert von größer als 50 mm
Verhaltensbeobachtung der PatientInnen inkl. dysfunktionaler Verhaltensweisen
Anwendung eines Angstfragebogens
Strukturiertes Interview zur Erhebung der Anamnese
Feststellung eines Vermeidungsverhaltens über mehr als 2 Jahre
*Klinische Untersuchung inkl. der orofazialen Funktion (falls trotz ZBA möglich)*
ZBA ohne Krankheitswert: Je nach Präferenz der PatientInnen optional unterstützende oder stressreduzierende Verfahren wie Musik, Entspannung, Lokalanästhesie
*Hinzuziehung von FachärztInnen oder psychologischer PsychotherapeutInnen zur Initiierung einer Verhaltenstherapie*
Bei pathologischen Formen der ZBA mit Krankheitswert, aber auch ZBA als Symptom anderer psychischer Störungen (z. B. andere Angst- oder Traumafolgestörungen): Verhaltenstherapie meist in 5 Sitzungen
Pharmakotherapie und weitere Interventionen

### ZBA und Parodontitis

Querschnittsstudien aus den USA zu Parodontitis, Zahnverlust und Inanspruchnahmeverhalten bestätigten eine Assoziation von ZBA und Depression [15]. Karies hingegen scheint eher durch sozioökonomische Faktoren beeinflusst zu werden [16]. In einer Metaanalyse aus 40 Fall-Kontroll-Studien wurde eine ausgeprägte Heterogenität der Studienergebnisse bei Erwachsenen festgestellt. ParodontitispatientInnen aus 18 Studien hatten vor der parodontalen Behandlung einen geringgradig signifikant höheren Depressionsscore und in 12 Studien einen erhöhten Angstscore [17]. Der initiale und langfristige Erfolg einer parodontalen Therapie hängt wesentlich von der Einsicht Betroffener hinsichtlich der Bedeutung der eigenen Adhärenz (Therapietreue) ab [18]. Die Information zur Notwendigkeit der aktiven und lebenslangen unterstützenden Parodontaltherapie sollte durch die Information über psychosoziale Unterstützungsangebote ergänzt werden [16]. Die Stärke des Sense of Coherence (SOC) als globaler Blick auf die Gesundheit und das patienteneigene positive Gefühl, das Leben auch während und mit der Behandlung gestalten zu können, scheint nicht mit einem besseren Behandlungsergebnis assoziiert [19]. PatientInnen mit höherem SOC nehmen mehr Behandlungsangebote wahr [20]. Die Darstellung der personalisierten psychosozialen Vorteile der parodontalen Therapie sind für Behandlungserfolge in allen Therapiephasen notwendig und verhindert die Ausbildung von ZBA mit Krankheitswert [21].

## KraniomandibuläreDysfunktion (CMD)

Unter einer CMD wird eine Reihe von klinischen Symptomen im Bereich der Kaumuskulatur und/oder der Kiefergelenke zusammengefasst. Die Leitsymptome sind Funktionseinschränkungen und/oder Schmerz als Kaumuskelschmerz und/oder Kiefergelenkschmerz und Schmerz als Folge von Bewegungsstörungen des Kauorgans (z. B. schmerzhaft eingeschränkte Mundöffnung oder Schmerzen beim Kauen). Die Beschwerden können akut auftreten, aber auch über einen längeren Zeitraum chronifizieren. In Abhängigkeit von der Intensität und Beeinträchtigung werden ZahnärztInnen und ÄrztInnen (z. B. HNO-ÄrztInnen bei Schmerzen im Bereich der Kiefergelenke) konsultiert. Der geschätzte Behandlungsbedarf bei 19- bis 45-Jährigen liegt bei etwa 16 % [22]. Frauen scheinen nach aktueller Datenlage häufiger betroffen zu sein [23]. Genannte Gründe dafür sind eine höhere Belastung durch Stress, eine exogene Hormonsubstitution sowie auch Zyklusschwankungen mit erhöhter Prävalenz für chronischen Schmerz [24]. Grundsätzlich wird eine multifaktorielle Genese im Sinne eines biopsychosozialen Krankheitsgeschehens als gesichert angenommen. Der Stellenwert der verschiedenen Faktoren ist hoch individuell und variabel und ihre genaue Pathologie im Einzelnen noch nicht abschließend geklärt. Unter psychopathologischer Sicht werden Angst, Depression, Somatisierungstendenzen und das Gefühl mangelnder Kontrolle hinsichtlich der eigenen Gesundheit sowie stärker erlebte Belastungen durch Alltagsstress bzw. eine mangelhafte Stressverarbeitung Schlüsselstellungen zuerkannt [25–27]. Es gibt Hinweise darauf, dass vor allem eine inadäquate Stressverarbeitung über das Kauorgan die Symptomatik aufrechterhält. Psychosoziale Auffälligkeiten können sowohl Ursache als auch Folge sein. Insbesondere bei chronisch bestehender Symptomatik lassen sich die Abhängigkeiten nicht immer eindeutig zuordnen.

### Diagnostik der CMD

Grundsätzlich gilt für die Diagnostik der CMD, dass alle Facetten der Erkrankung, d. h. die somatischen Symptome und Störungen ebenso wie mögliche psychosoziale Einflussfaktoren, frühzeitig erfasst werden müssen. Erste Hinweise auf das Vorliegen somatischer Symptome einer CMD sollten mithilfe von symptomorientierten Screenings überprüft werden. Diese stehen kostenfrei über die Homepage der Deutschen Gesellschaft für Funktionsdiagnostik und -therapie (DGFDT) zur Verfügung [28] Aus juristischer Sicht sind solche Screenings auch bei Nichtvorliegen von Symptomen und insbesondere vor prothetischer Therapie unabdingbar. Dieses geht aus einer „... oberlandesgerichtlichen Rechtsprechung (OLG Hamm, Urteil vom 04.07.2014, Az.: 26 U 131/13 und OLG München, Urteil vom 18.01.2017, Az.: 3 U 5039/13) hervor, mit der klargestellt wird, dass eine prothetische Therapie ohne vorheriges CMD-Screening zur Abklärung einer unentdeckten CMD einen Behandlungsfehler darstellt“ [29]. Sofern sich auf Basis des somatischen Screenings Hinweise auf eine CMD ergeben, kann eine umfassende Funktionsdiagnostik eingeleitet werden [30]. Die psychosozialen Aspekte und mögliche Chronifizierungstendenzen sollten gleichfalls initial erfasst werden. Dabei ist zu bedenken, dass in dem somatischen Umfeld einer zahnärztlichen Praxis initial Screenings mit somatischem Bezug eingesetzt werden sollten. Screenings, die ohne Bezug auf die Symptomatik und entsprechende Erklärung ausschließlich psychosoziale Aspekte abfragen, werden von PatientInnen als unangemessen bewertet und abgelehnt, da sie mit einer anderen Erwartungshaltung die zahnärztliche Sprechstunde aufsuchen. Somit bietet sich zur Feststellung einer Chronifizierung der Graded-Chronic-Pain(GCP-)Status an [31], in welchem bei allen Fragen immer der Bezug zur Symptomatik betont wird. Somatisierungstendenzen lassen sich mit Fragebögen zu körperlichen Beschwerden evaluieren (z. B. Beschwerdenliste nach von Zerssen; [32]). Bei anamnestischen Hinweisen auf Angst und/oder Depressionen können entsprechende psychologische Frageinstrumente (z. B. Hospital Anxiety and Depression Scale (HADS) [33]) eingesetzt werden. Diese sollten jedoch im Vorfeld erläutert werden, um die notwendige Compliance zur ehrlichen Beantwortung zu erlangen.

### Therapie der CMD

Im Mittelpunkt der Therapie steht die Aufklärung über die aufgezeigten Zusammenhänge und die möglicherweise fehlende Selbstkontrolle [34]. Eine verbesserte Selbstkontrolle können PatientInnen bei Bedarf durch gezielte Verhaltensänderung (ggf. unterstützt durch Biofeedback) erlernen und somit Stress ohne Gesundheitsschädigung verarbeiten. Beim Biofeedback werden körperliche Funktionen optisch und akustisch kontinuierlich rückgemeldet und positive Veränderungen dieser Körperfunktionen verstärkt. Auf diese Weise soll ein positiv verstärkter Lernprozess initiiert werden [35] mit dem Ziel einer verbesserten Eigenkontrolle und damit im gewünschten Sinne möglichen Korrektur. Sofern erforderlich kann für längerfristige Verhaltensänderungen eine entsprechende kognitive Verhaltenstherapie angeboten werden [34, 36]. Zur Behandlung der körperlichen Symptomatik sollten reversible Maßnahmen eingesetzt werden. Dazu gehört die Anfertigung einer Aufbissschiene als Schutz zum Erhalt der Zahnhartsubstanz. Gute Effekte zeigen physiotherapeutische Maßnahmen und die Anwendung von Biofeedback [37, 38].

## Bruxismus

Im Jahr 2013 wurde Bruxismus entsprechend einem internationalen Expertenkonsens definiert als „eine wiederholte Kaumuskelaktivität, charakterisiert durch Kieferpressen und Zähneknirschen und/oder Anspannen oder Verschieben des Unterkiefers ohne Zahnkontakt“ [3, 39]. Die Folge intensiven Knirschens mit den Zähnen sind Zahnhartsubstanzverluste (Attritionen = intrinsische mechanische Abnutzung in Funktion und/oder bei Parafunktionen (z. B. Bruxismus) aufgrund gegenseitigen Kontaktes der Zähne). „Bruxismus kommt in zwei zu unterscheidenden zirkadianen Erscheinungsformen vor: er kann während des Schlafs auftreten (Schlafbruxismus = SB) und während des Wachseins (Wachbruxismus = WB). … SB wird charakterisiert als rhythmisch (phasisch) oder nicht-rhythmisch (tonisch) und ist keine Bewegungsstörung oder eine Schlafstörung bei ansonsten gesunden Individuen. … WB ist eine Aktivität der Kaumuskulatur während des Wachzustands. WB wird charakterisiert als wiederholter oder dauerhafter Zahnkontakt und/oder als Anspannen oder Verschieben des Unterkiefers ohne Zahnkontakt. Bei ansonsten gesunden Individuen handelt es sich dabei nicht um eine Bewegungsstörung“ [3]. Die Prävalenzzahlen variieren zwischen 22,1–31 % bei WB und 12,8 % ± 3,1 % bei SB. Eine geschlechterabhängige Prävalenz lässt sich nicht eindeutig nachweisen [43].

Die Ätiologie des Bruxismus ist multifaktoriell, wobei sich die Aufmerksamkeit zunehmend auf Faktoren wie Stress (selbst wahrgenommen oder im Sinne einer negativen Stressverarbeitung), Angststörungen, Depressivität, psychosoziale Störungen, Schlafstörungen (z. B. Insomnie), physiologische/biologische/genetische Faktoren, neurochemische Transmitter, Reflux oder exogene Faktoren, wie Nikotin‑, Alkohol- oder Drogenkonsum, richtet. Die in der Vergangenheit mit hoher Relevanz bewerteten peripheren Faktoren (z. B. Okklusionsstörungen) werden aktuell allenfalls als sekundäre Einflussfaktoren eingestuft [3].

### Diagnostik des Bruxismus

Sowohl berichtete Symptome als auch Zufallsbefunde können auf Bruxismus hinweisen (Tab. 2). Die Folge von Bruxismus im Sinne von Zähneknirschen ist ein irreversibler Zahnhartsubstanzverlust. Um die durch Bruxismus verursachten langfristigen funktionellen, ästhetischen und dentalen Folgen zu vermeiden, ist eine frühzeitige Diagnostik entscheidend. Hinweise auf Bruxismus können, sofern dieser nicht selbst berichtet wird, mithilfe eines Screenings festgestellt werden [40]. Bei Auffälligkeiten im Screening ist zur weiteren Diagnostik eine ausführliche Anamneseerhebung erforderlich, ggf. auch unter Einbeziehung von PartnerInnen, die z. B. nächtliches Zähneknirschen wahrnehmen. Sofern entsprechende dentale und/oder muskuläre Befunde anamnestische Angaben nicht eindeutig belegen, kann das vorübergehende Führen eines „Bruxismus-Beschwerdetagebuchs“ unterstützend eingesetzt werden. Zur Evaluation der Aspekte Angst, Depression, Stressverarbeitung können Fragebögen vergleichbar mit denen der CMD-Diagnostik verwendet werden. Hier bedarf es im Vorfeld ebenfalls der entsprechenden Aufklärung, um Irritationen angesichts einer zahnmedizinischen Diagnostik- und Therapieerwartung zu vermeiden. Weitere apparative Verfahren beschreibt und bewertet die S3-Leitlinie „Diagnostik und Behandlung von Bruxismus“ [3].

**Table 2:** 

*Anamnese und Symptome*
Schmerzen im Bereich der Kiefergelenke, Kau- und/oder Nackenmuskulatur
Kopfschmerz
Auffällige Empfindlichkeit der Zähne oder Zahnlockerungen ohne parodontale Probleme
Schlechte Schlafqualität
*Klinische Besonderheiten*
Zahnhartsubstanzverlust ohne Kariesbefall/Abplatzungen bzw. Schäden an Zahnersatzmaterialien
Zungen‑/Wangenimpressionen, weißliche Wangenlinie
Hypertrophe Kaumuskulatur
Reduzierte Mundöffnungsbeweglichkeit

### Therapie des Bruxismus

Die Aufklärung über Ursache und Folgen des Bruxismus nimmt in der Therapie den zentralen Stellenwert ein. Dazu zählt der Hinweis auf möglicherweise bis zu diesem Zeitpunkt nicht wahrgenommene Muskelaktivität/Parafunktionen ebenso wie die Beruhigung, sofern Betroffene durch klinische Veränderungen unverhältnismäßig beunruhigt sind (Katastrophisierungstendenzen; [41]). Auf diese Weise kann eine Verhaltensänderung eingeleitet werden. Zum kontrollierten Stressabbau eignet sich der Einsatz eines Biofeedbacks. Die bisherige Datenlage weist darauf hin, dass beide Bruxismusformen durch das Biofeedback beeinflusst werden können. Umstritten ist der Einsatz von akustischen Feedbacksignalen zur Therapie von SB, da das Signal den Nachtschlaf stört und eine beeinträchtigende Tagesmüdigkeit zur Folge haben kann [42]. Physiotherapie eignet sich bei körperlichen Beschwerden und im Sinne der Anleitung zur Selbsthilfe. Abgesehen von der Behandlung akuter Schmerzzustände ist eine medikamentöse Therapie nicht empfohlen. Zum Schutz vor Attritionen sollten Aufbissschienen eingesetzt werden [43].

## Okklusale Dysästhesie (OD)

Die OD bezeichnet Erkrankungen bei PatientInnen, die im Wachzustand über Beschwerden ausgehend von der Okklusion (= Zubiss) klagen. Zahnmedizinische Ursachen dafür lassen sich nicht bzw. nicht hinreichend objektivieren. In der Regel lassen sich eine psychosoziale Belastung und auch Merkmale einer somatischen Belastungsstörung^1^, Persönlichkeitsstörung, Zwanghaftigkeit, Depression und/oder Angststörung feststellen.

Die OD kennzeichnet eine nahezu ausschließliche Fokussierung auf eine somatische/okklusale Ursache des Leidens im Sinne einer Zubissstörung und erhebliche Gewöhnungsstörungen nach zahnmedizinischen Maßnahmen. Jeder Versuch, durch somatische Behandlung (z. B. dem Einschleifen des Zubisses) Linderung zu verschaffen, führt in der Regel zu einer Intensivierung. Das für dieses Krankheitsbild in der Literatur beschriebene mittlere Alter wird mit 52 Jahren (plus/minus 11 Jahre) angegeben. In den Zeitraum der Erstmanifestation fallen häufig belastende Lebensereignisse. Frauen sind etwa 5‑mal häufiger betroffen. Zumeist kann man von einer Chronifizierung der Beschwerden ausgehen. Als ätiologische Faktoren werden psychopathologische Ursachen, Neuroplastizität, Phantomphänomene und Veränderungen der propriozeptiven Reize und ihrer Übertragung diskutiert [1].

### Diagnostik der OD

Es besteht eine deutlich erkennbare Diskrepanz zwischen klinischem Befund (differenzialdiagnostische Abgrenzung zur Okklusopathie im Sinne einer objektivierbaren Zubissstörung) und dem erheblichen Ausmaß subjektiver Beschwerden. Die Anamnese ist gekennzeichnet durch vielfache diagnostische Maßnahmen und Therapieversuche. Die S1-Leitlinie empfiehlt die folgenden Befundbögen zur Evaluation psychischer (Co‑)Faktoren [1]:

Chronifizierung: Graded Chronic Pain Score (GCPS); Angst, Depression: HADS; Emotionaler Stress: Social Readjustment Rating Scale (SRRS); Somatisierung: Befindlichkeitsskala – Revidierte Fassung (BL-R/BL-R’); Schmerzlokalisationen: Ganzkörperzeichnung aller bestehenden Schmerzbereiche.

### Therapie der OD

Es ist grundsätzlich *nicht* empfehlenswert, die OD durch okklusale Maßnahmen im Sinne einer Zubissveränderung/-korrektur zu therapieren. Durch übermäßige zahnmedizinische/okklusale Therapieversuche kommt es in der Regel zu einer weiteren iatrogen somatischen Fixierung und somit therapieresistenten Chronifizierung. Ziel jeder Maßnahme sollte es sein, dass Betroffene lernen, okklusale Signale adäquat einzuordnen (Defokussierung), Reize (ggf. durch den temporären Einsatz einer Aufbissschiene) zu reduzieren und die Lebensqualität mit angemessener Kontrolle eigener Emotionen zu verbessern. Als medikamentöse Therapie kann bei entsprechender Indikationsstellung zum Beispiel die Gabe von Amitriptylin, Mirtazapin oder Aripiprazol in Erwägung gezogen werden.

## Somatoforme Prothesenunverträglichkeit (SP)

Neben dem Burning Mouth Syndrome (BMS), der somatoformen Schmerzstörung und der körperdysmorphen Störung (als Sonderform) stellt die SP eine für die Zahnmedizin relevante Untergruppe der somatoformen Störung dar: „Das Charakteristikum der somatoformen Störung ist die wiederholte Darbietung körperlicher Symptome in Verbindung mit hartnäckigen Forderungen nach Untersuchungen trotz wiederholter negativer Ergebnisse und Versicherungen der Ärzte, dass die Symptome nicht körperlich begründbar sind. Ist aber irgendeine Organpathologie vorhanden, dann erklärt sie nicht die Art und das Ausmaß der Symptome oder das Leiden und die innerliche Beteiligung des Patienten“ [2]. Der Begriff „Prothesenunverträglichkeit“ kann zu 2 Missverständnissen führen. Zum einen unterstellt er fälschlicherweise eine Unverträglichkeit im Sinne einer Materialunverträglichkeit. Zum anderen wird suggeriert, dass es sich bei dem Phänomen ausschließlich um Beschwerden mit herausnehmbarem Zahnersatz handelt. Das mag vor dem Hintergrund der anfänglichen Auseinandersetzung mit dieser Thematik auch so gewesen sein, da die PatientInnen in der Regel älter als 55 Jahre und mit herausnehmbarem Zahnersatz versorgt waren. Heute sind zunehmend PatientInnen mit allen Versorgungsarten davon betroffen. Dennoch ist der Begriff der „Prothesenunverträglichkeit“ nicht falsch vor dem Hintergrund der eigentlichen Bedeutung des Begriffes „Prothese“: künstlicher Ersatz eines fehlenden Körperteils. In diesem Sinne ist damit jegliche Ersatzform einbezogen. Die Betroffenen klagen in der Regel über einen sehr langen Zeitraum (> 6 Monate) über Schmerz, Mundschleimhautbrennen und/oder Gewöhnungsstörungen. Begleitet werden diese Leitsymptome teils von Mundtrockenheit oder Geschmacksirritationen. Die Beschwerden sind hinsichtlich ihrer Intensität individuell unterschiedlich und variabel. Sie können lokalisiert auftreten oder auch weit über die Mundhöhle hinaus den gesamten Körper betreffen. Die Betroffenen sind der festen Überzeugung, dass die Ursache allen Leidens im Zahnsystem verankert ist und auch nur eine entsprechende Zahnbehandlung Heilung verschaffen wird. Der Weg der PatientInnen auf der Suche nach Entlastung ist somit gekennzeichnet von „Ärztehopping“. Betroffene wie auch nahestehende Personen unterwerfen das gesamte Leben dem Diktat der Beschwerden und die Lebensqualität ist deutlich reduziert [44]. Diese beschriebenen Merkmale sind auch wesentliche Kriterien der neu in die Klassifikationen eingeführten Diagnose der „somatischen Belastungsstörung“, was somit als übergeordnete Kategorie angesehen werden kann.

### Diagnose und Differenzialdiagnose der SP

Parallel zur psychosozialen Diagnostik sollte die SP in jedem Fall gründlich nicht redundant somatisch diagnostiziert werden, ggf. auch interdisziplinär. Eine rein somatische Ausschlussdiagnostik ist nicht gerechtfertigt. Kriterien zum Erkennen einer solchen Störung hat Müller-Fahlbusch [45] formuliert. Diese sind vor allem die Diskrepanz zwischen Beschreibung der Beschwerden und anatomischen Grenzen, die Diskrepanz zwischen Chronologie der Beschwerden und den uns aus klinischer Erfahrung bekannten Verläufen sowie auffällig viele subjektiv gescheiterte, aber objektiv suffiziente Therapieversuche. Darüber hinaus fällt die ungewöhnliche Mitbeteiligung und das Leiden am Krankheitsgeschehen auf und eine vielfach nachvollziehbare Koinzidenz von Beschwerdebeginn und be- bzw. entlastenden Lebenssituationen [45].

Differenzialdiagnostisch abgegrenzt werden muss das Mundschleimhautbrennen, ein Leitsymptom der SP. Das Krankheitsbild des Mundschleimhautbrennens wird sehr heterogen definiert, weshalb Angaben aus Studien zu diesem Phänomen sehr variieren. Im Sinne der Definition von Scala et al. [46] unterscheidet man das primäre/idiopathische und dann bezeichnete BMS von einem sekundären Mundschleimhautbrennen als Folge einer feststellbaren zahnmedizinischen, medizinischen und/oder psychischen Ursache [47]. Das BMS basiert auf einer Ausschlussdiagnostik, was so auch in neueren Definitionen beschrieben wird [48]. Betroffen sind eher Frauen ab dem 50. Lebensjahr. Da viele Studien BMS nicht immer gleich definieren, lässt sich bezogen auf psychische Faktoren nicht durchgängig unterscheiden, ob diese Ursache für ein sekundäres Mundschleimhautbrennen sind oder infolge eines (idiopathischen) BMS auftreten. Genannt werden im Kontext des Mundschleimhautbrennens Depression, Somatisierungsstörungen und Angstzustände bis hin zur Krebsphobie, was bei ca. 20 % der BMS-PatientInnen zu beobachten ist [44].

### Therapie der SP

Ähnlich wie bei der OD sollten (und müssen in vielen Fällen) nur streng lokal indizierte Maßnahmen erfolgen. Allein beschwerdegesteuerte somatische/zahnmedizinische Therapien sind kontraindiziert. Umfassende Aufklärung im Sinne eines biopsychosozialen Krankheitsgeschehens und das Aufzeigen der Prognose bei weiteren irreversiblen somatischen Behandlungsversuchen sind die Grundlage, um ein Verständnis für eine ggf. interdisziplinäre Therapieform unter Einbeziehung psychosomatischer Fachdisziplinen zu erreichen. Da ZahnarztpatientInnen in der Regel nicht direkt in andere medizinische Fachdisziplinen überwiesen werden, ist eine gründliche Aufklärung über die Zusammenhänge der Erkrankung unabdingbar. Nur so können Betroffene ausreichend motiviert werden, mit ihrem Anliegen die hausärztliche Praxis aufzusuchen.

## Fazit

Zusammenfassend lässt sich feststellen, dass psychosoziale Zusammenhänge in der Zahnmedizin eine große Rolle spielen können und somit bedacht werden sollten. Eine entsprechende Diagnostik ist in der Regel zeitintensiv, vermeidet aber eine für alle Beteiligten hoch belastende Situation bei nicht angemessener Vorgehensweise sowie Über- oder Untertherapien. Nachhaltige Behandlungsformen zur Angstreduktion sollen weiterentwickelt werden. Leitlinien zur ZBA, zum Bruxismus und zur OD bieten wissenschaftlich fundierte Informationen über Diagnose- und Therapiemöglichkeiten. Die darin enthaltenen Patientenleitlinien sind gerade für „Nichtzahnärzte/innen“ hilfreich bei der Aufklärung Betroffener. ZahnärztInnen haben seit 2006 die Möglichkeit, ein Curriculum zur „Psychosomatischen Grundkompetenz“ der Akademie Praxis und Wissenschaft zu absolvieren. Mit Einführung der neuen Approbationsordnung für ZahnärztInnen 2021 werden unter anderem Aspekte wie Patientenführung, Arzt-Patienten-Gespräch und Psychologie in den Lehrplan aufgenommen. Zur Sensibilisierung nichtzahnmedizinischer Fachdisziplinen für diese spezifischen zahnmedizinischen Themen werden in Lehrbücher und Curricula zur Stressmedizin und Schmerztherapie zunehmend auch relevante zahnmedizinische Themen integriert. In den allgemeinmedizinischen Disziplinen sollte bedacht werden, dass ZahnärztInnen vielfach nicht direkt überweisen können. Stattdessen können sie die PatientInnen nur motivieren, die hausärztliche Praxis aufzusuchen und ggf. dort das Anliegen zu besprechen. Damit ist die Gefahr besonders bei PatientInnen mit somatischer Fixierung groß, dass die Intention nicht entsprechend übermittelt wird. Damit dennoch eine Kommunikation zwischen ZahnärztInnen und spezialisierten FachkollegInnen stattfindet, ist es entscheidend, dass einerseits von zahnmedizinischer Seite alle relevanten Informationen schriftlich übermittelt werden. Andererseits sollte darauf geachtet werden, dass im Gegenzug alle relevanten Informationen auch den ZahnärztInnen zugänglich sind. Auf diese Weise wird sichergestellt, dass der Kenntnisstand der unterschiedlichen Behandelnden gleich ist und damit oft unbewusste, aber dennoch kontraproduktive „Manipulationen“ seitens der PatientInnen vermieden werden.

## Footnotes

1 Somatische Belastungsstörung: Unabhängig davon, ob eine somatische Ursache zugrunde liegt oder nicht, beschreibt die somatische Belastungsstörung die individuellen Auswirkungen der Beschwerden. Diese Diagnose ist gerechtfertigt auf Basis einer Symptomdauer von mehr als 6 Monaten, der auffälligen Beschäftigung mit den Beschwerden auf kognitiver, emotionaler und Verhaltensebene sowie der Störung des Alltagslebens.

## References

[1] AWMF (2019) S1-Leitlinie: Okklusale Dysästhesie – Diagnostik und Management, AWMF-Registernummer: 083-037. https://www.awmf.org/leitlinien/detail/ll/083-037.html. Zugegriffen: 8. Jan. 2021

[2] Doering S, Wolowski A (2008) Psychosomatik in der Zahn‑, Mund- und Kieferheilkunde. Wissenschaftliche Mitteilung des Arbeitskreises Psychologie und Psychosomatik in der DGZMK. https://secure.owidi.de/documents/10165/2216111/Psychosomatik_in_der_Zahn-_Mund-_und_Kieferheilkunde_2008.pdf/0f0bbb61-d371-490d-b22d-65d6865976e4. Zugegriffen: 8. Jan. 2021

[3] AWMF (2019) S3-Leitlinie (Langversion) Diagnostik und Behandlung von Bruxismus, AWMF-Registernummer: 083-027. https://www.dgfdt.de/documents/266840/3732791/Leitlinie+Bruxismus/40b51e33-c45e-49a6-80fd-0889132e8aaf. Zugegriffen: 8. Jan. 2021

[4] Manfredini D, Winocur E, Guarda-Nardini L, Paesani D, Lobbezoo F (2013). Epidemiology of bruxism in adults: a systematic review of the literature. J Orofac Pain.

[5] de Zwaan M, Müller A (2006). Über den Umgang mit schwierigen Patientinnen und Patienten. Wien Med Wochenschr.

[6] Braas R, Eger T, Gohr J, Wörner F, Wolowski A (2019). Orofacial dysfunction and posttraumatic stress disorder: a context analysis of soldiers after military deployment. Nervenarzt.

[7] Jacobi F, Wittchen HU, Hölting M, Höfler M, Pfister H, Müller N (2004). Prevalenz, comorbidity and correlates of mental disorders in the general population. Results from German Health Interview and Examination Survey (GHS). Psychol Med.

[8] AWMF (2019) S3 Leitlinie, Zahnbehandlungsangst beim Erwachsenen, AWMF-Registernummer: 083-020. http://www.awmf.org/uploads/tx_szleitlinien/83-020I_S3_Zahnbehandlungsangst-beim-Erwachsenen_2019-11.pdf. Zugegriffen: 20. Jan. 2021

[9] Pohjola V, Mattila AK, Joukamaa M, Lahti S (2011). Dental fear and alexithymia among adults in Finland. Acta Odontol Scand.

[10] Eger T, Wörner F, Simon U, Konrad S, Wolowski A (2021). Dental anxiety and higher sensory processing sensitivity in a sample of German soldiers with inflammatory periodontal disease. Int J Environ Res Public Health.

[11] Lenk M, Berth H, Joraschky P, Petrowski K, Weidner K, Hannig C (2013). Fear of dental treatment—an underrecognized symptom in people with impaired mental health. Dtsch Arztebl Int.

[12] Joehren P (1999). Validierung eines Fragebogens zur Erkennung von Zahnbehandlungsangst. Dtsch Zahnarztebl.

[13] Enkling N, Marwinski G, Jöhren P (2006). Dental anxiety in a representative sample of residents of a large German city. Clin Oral Investig.

[14] Wannemueller A, Joehren HP, Borgstaedt A (2017). Large group exposure treatment: a feasibility study of exposure combined with diaphragmatic breathing in highly dental fearful individuals. Front Psychol.

[15] Aldosari M, Helmi M, Kennedy EN (2020). Depression, periodontitis, caries and missing teeth in the USA, NHANES 2009–2014. Fam Med Community Health.

[16] Ehrenthal JC, Graetz C, Plaumann A, Dörfer CE, Herzog W (2016). Number of teeth predict depressive symptoms in a longitudinal study on patients with periodontal disease. J Psychosom Res.

[17] Zheng DX, Kang XN, Wang YX (2021). Periodontal disease and emotional disorders: a meta-analysis. J Clin Periodontol.

[18] Machado V, Botelho J, Proença L, Mendes JJ (2020). Self-reported illness perception and oral health-related quality of life predict adherence to initial periodontal treatment. J Clin Periodontol.

[19] Bertoldi C, Venuta M, Guaraldi G (2018). Are periodontal outcomes affected by personality patterns? An 18-month follow-up study. Acta Odontol Scand.

[20] Holde GE, Baker SR, Jönsson B (2018). Periodontitis and quality of life: What is the role of socioeconomic status, sense of coherence, dental service use and oral health practices? An exploratory theory-guided analysis on a Norwegian population. J Clin Periodontol.

[21] Horne PE, Page LAF, Leichter JW, Knight ET, Thomson WM (2020). Psychosocial aspects of periodontal disease diagnosis and treatment: a qualitative study. J Clin Periodontol.

[22] Al-Jundi MA, John MT, Setz JM, Szentpetery A (2008). Meta-analysis of treatment need for temporomandibular disorders in adult nonpatients. J Orofac Pain.

[23] Gesch D, Bernhardt O, Alte D, Schwahn C, John U, Hensel E (2004). Prevalence of signs and symptoms of temporomandibular disorders in an urban and rural German population: results of a population-based study of health in Pomerania. Quintessence Int.

[24] LeResche L, Mancl L, Sherman JJ, Gandara B, Dworkin SF (2003). Changes in temporomandibular pain and other symptoms across the menstrual cycle. Pain.

[25] Bonjardim LD, Duarte Gavião MB, Pereira LJ, Castelo MP (2005). Anxiety and depression in adolescents and their relationship with signs and symptoms of temporomandibular disorders. Int J Prosthodont.

[26] Glaros AG, Burton E (2004). Parafunction, clenching, pain in effort in temporomandibular disorders. J Behav Med.

[27] Reissmann DR, John MT, Schierz O, Wassell RW (2007). Functional and psychosocial impact related to specific temporomandibular disorder diagnoses. J Dent.

[28] DGFDT (2020) CMD-Screening (CMD-Basisdiagnostik) der DGFDT. https://www.dgfdt.de/documents/266840/22655647/CMD-Screening+2020/9546df75-5798-4a84-871d-2d16742e38bc. Zugegriffen: 18. März 2021

[29] Peroz I, Ahlers MO, Hugger A (2020). CMD-Screening ist wichtig. Schmerz.

[30] DGFDT (2021) Befundbögen auf Homepage der DGFDT. https://www.dgfdt.de/richtlinien_formulare. Zugegriffen: 18. März 2021

[31] Türp JC, Schindler HJ (2006). Myoarthropathien des Kausystems: X – Diagnostik: Graduierung chronischer Schmerzen. Zahnarztl Prax.

[32] Von Zerssen D, Petermann F (2011). Befindlichkeitsskala – Revidierte Fassung.

[33] Herrmann C, Buss U (1994). Vorstellung und Validierung einer deutschen Version der „Hospital Anxiety and Depression Scale“ (HAD-Skala). Ein Fragebogen zur Erfassung des psychischen Befindens bei Patienten mit körperlichen Beschwerden. Diagnostica.

[34] Michelotti A, Steenks MH, Farella M (2004). The additional value of a home physical therapy regimen versus patient education only for the treatment of myofascial pain of the jaw muscles: short-term results of a randomized clinical trial. J Orofac Pain.

[35] Rief W, Birbaumer N, Rief W, Birbaumer N (2011). Grundsätzliches zu Biofeedback. Biofeedback. Grundlagen, Indikationen, Kommunikation, Vorgehen.

[36] Crider AB, Glaros AG, Gevitz RN (2005). Efficacy of biofeedback-based treatments for temporomandibular disorders. Appl Psychophysiol Biofeedback.

[37] van Grootel RJ, Buchner R, Wismeijer D, van der Glas HW (2017). Towards an optimal therapy strategy for myogenous TMD, physiotherapy compared with occlusal splint therapy in an RCT with therapy-and-patient-specific treatment durations. BMC Musculoskelet Disord.

[38] Florjanski W, Malysa A, Orzeszek S, Smardz J, Olchowy A, Paradowska-Stolarz A (2019). Evaluation of biofeedback usefulness in masticatory muscle activity management—a systematic review. J Clin Med.

[39] Lobbezoo F, Ahlberg J, Raphael KG (2018). International consensus on the assessment of bruxism: report of a work in progress. J Oral Rehabil.

[40] DGFDT (2019) Bruxismus Screening Index (BSI) der DGFDT. https://www.dgfdt.de/documents/266840/3732097/BSI+zweiseitig+2020/dad1b99a-c1a8-4f58-a85c-1df897176466. Zugegriffen: 18. März 2021

[41] Panek H, Nawrot P, Mazan M (2012). Coincidence and awareness of oral parafunctions in college students. Community Dent Health.

[42] Lobbezoo F, Aarab G, Ahlers MO (2020). Consensus-based clinical guidelines for ambulatory electromyography and contingent electrical stimulation in sleep bruxism. J Oral Rehabil.

[43] Manfredini D, Ahlberg J, Winocur E, Lobbezoo F (2015). Management of sleep bruxism in adults: a qualitative systematic literature review. J Oral Rehabil.

[44] Wolowski A (2021). Ist der Begriff der Prothesenunverträglichkeit noch zeitgemäß?. Dtsch Zahnärztl Z.

[45] Müller-Fahlbusch H (1992). Ärztliche Psychologie in der Zahnmedizin.

[46] Scala A, Checchi L, Montevecchi M, Marini I (2003). Update on burning mouth syndrome: Overview and patient management. Crit Rev Oral Biol Med.

[47] Wolowski A, Runte C (2013). Somatische Reaktionen nach restaurativer Therapie – somatisches oder psychosomatisches Krankheitsbild?. Dtsch Zahnärztl Z.

[48] International Headache Society (2020). International Classification of Orofacial Pain (ICOP). Cephalalgia.

